# Best evidence summary for preventing and preventing epidermal growth factor receptor inhibitors induced paronychia in cancer patients

**DOI:** 10.3389/fonc.2025.1555114

**Published:** 2025-04-14

**Authors:** Kexuan Li, Xiuyue Qiu, Jiayu Zhang, Yingshuyi Zhou, Youfen Fan

**Affiliations:** ^1^ Burn Department, Ningbo No.2 Hospital, Ningbo, Zhejiang, China; ^2^ School of Nursing, Zhejiang Chinese Medical University, Hangzhou, Zhejiang, China; ^3^ Nursing Department, Ningbo No.2 Hospital, Ningbo, Zhejiang, China

**Keywords:** cancer, target therapy, epidermal growth factor receptor inhibitors, paronychia, evidence summary

## Abstract

**Objective:**

To evaluate and summarize the best evidence for preventing and managing epidermal growth factor receptor inhibitors induced paronychia in cancer patients. It aims to provide a reference for medical staff.

**Methods:**

We systematically searched for evidence on paronychia symptoms in 14 databases such as Cnki、Wanfang, 7 guide websites such as GIN and NZGG, and 8 professional websites such as UICC and ACS from database establishment to August 2024. Two researchers evaluated the quality of the literature and extracted the data.

**Results:**

A total of 20 articles were included in this study, including 5 clinical decisions、5 guidelines、3 evidence summaries 、3 recommended practices, and 4 experts consensuses. Finally, 12 pieces of evidence were summarized from 4 aspects, including risk factor assessment, professional healthcare training, preventive measures, and therapeutic measures.

**Conclusion:**

Our research summarizes the best evidence for preventing and managing epidermal growth factor receptor inhibitors induced by paronychia in cancer patients. In the actual clinical application, it is necessary to fully consider the clinical situation, combine the judgment of professionals and patients’ wishes, follow the principle of individualization, analyze the obstacles and facilitating factors of the application of evidence, and apply the evidence to the clinical practice prudently.

## 1 Introduction

Epidermal growth factor receptor (EGFR) is a transmembrane glycoprotein composed of an extracellular ligand-binding region and an intracellular domain containing tyrosine kinase activity (1). EGFR is overexpressed in various cancers, such as non-small cell lung cancer, pancreatic cancer, and colorectal cancer, which is currently one of the most common cancer-driver genes (2, 3). Epidermal growth factor receptor inhibitors (EGFRIs) are commonly used target drugs that regard EGFR as an intervention target. EGFRIs selectively antagonize key signaling pathways for tumor growth and survival and control tumor cell proliferation, migration, adhesion, and angiogenesis. It finally induces tumor cell apoptosis by curbing its biological activity (4). Although EGFRIs can antagonize cancer progression, they have substantial drug toxicity. They can arrest keratinocyte growth and differentiation, thinning the periungual epidermis and perforation and shedding of the plate, followed by inflammatory reactions (5). EGFRIs-related nail changes mainly include changes in the nail plate, nail bed, and periungual tissue, of which paronychia is the most common, with a symptom incidence of 4 to 56.8% (6). Even though paronychia caused by EGFRIs does not endanger the lives of cancer patients, the daily activities of cancer patients are often significantly limited by pain. Cancer patients often dare not walk on the ground due to severe pain, causing adverse events such as falls. Some patients experience adverse outcomes such as medication delay, dose change, and medication cessation due to intolerance (7). Currently, clinical medical staff needs a systematic and in-depth understanding of paronychia caused by EGFRIs. Furthermore, their diagnosis, treatment, and nursing abilities still need to be improved. Therefore, this study aims to provide a reference for clinical medical workers by extracting and summarizing the best current evidence on preventing and treating paronychia caused by EGFRIs.

## 2 Materials and methods

### 2.1 Retrieval strategy

Two researchers with a background in evidence-based medicine employ the 6S evidence model, a hierarchical framework that categorizes evidence into six levels. This model is intended to assist medical professionals in the swift retrieval, assessment, and application of clinical evidence, thereby establishing a scientific basis for clinical decision-making. The evidence retrieval time was from database establishment to August 2024. The Chinese search keywords were “Cancer/tumor” “target/epidermal growth factor” “side effects/adverse events/toxicities/fingernails/toenails/periungual/paronychia/incarcerated nail”. The English search words were “tumor/cancer/neoplasm/oncology/carcinoma”“ “target/epidermal growth factor*/EGF*”“ “side effects/adverse reactions/adverse events/toxicity/nail/periungual lesions/periungual disease/paronychia”. The databases were UpToDate, BMJ, Zynx, DynaMed, Cochrane, Joanna Briggs Institute Library, CINAHL, Web of Science, PubMed, Embase, CBM, Cnki, Wanfang, and VIP. Guidance websites included WHO, Medlive, American Society of Clinical Oncology (ASCO), Scottish Intercollegiate Guidelines Network (SIGN), Institute for Clinical and Economic Review (ICER), New Zealand Guidelines Group (NZGG), National Institute for Health and Care Excellence (NICE), Guidelines International Network (GIN), National Guideline Clearinghouse (NGC). Professional Society websites included European Society of Medical Oncology (ESMO), National Cancer Institute (NCI), Union for International Cancer Control (UICC), American Cancer Society (ACS), Registered Nurses Association of Ontario (RNAO), and International Council of Nurses (ICN).

### 2.2 Inclusion and exclusion criteria

The inclusion criteria for literature were as follows: (1) The subjects were patients aged ≥18 years and underwent EGFRIs. (2) The study was related to the prevention and treatment of paronychia. (3) The types of literature included clinical decisions, guidelines, evidence summaries, recommended practices, and expert consensus. (4) The publication language was Chinese or English.

The exclusion criteria for literature were as follows: (1) The researchers could not obtain the full text, or an article was incomplete. (2) It was published repeatedly. (3) It was a conference report. (4) It was an unreasonable study design and low quality.

### 2.3 Criteria for literature quality evaluation

The evaluation criteria were selected according to the type of literature for quality evaluation. Criteria for assessing guideline quality were derived from the Appraisal of Guidelines for Research and Evaluation Instrument (AGREE II) (8). The JBI expert consensus quality assessment tool (2016 version) (9) was used to evaluate the included expert consensus. Clinical decisions, evidence summaries, and recommended practices were assessed by tracing the references and evaluating the quality according to the original study corresponding to the extracted evidence items.

### 2.4 Evidence quality evaluation

Two researchers independently completed the quality evaluation of the literature. Conflicts of evaluation opinions between the researchers were resolved through a third investigator’s discussion or evaluation of the literature. When evidence conclusions from different sources were repeated or conflicting, researchers followed the inclusion principle of prioritizing evidence-based, high-quality, and more recently published evidence.

## 3 Results

### 3.1 General characteristics of the included literature

**Figure 1:**
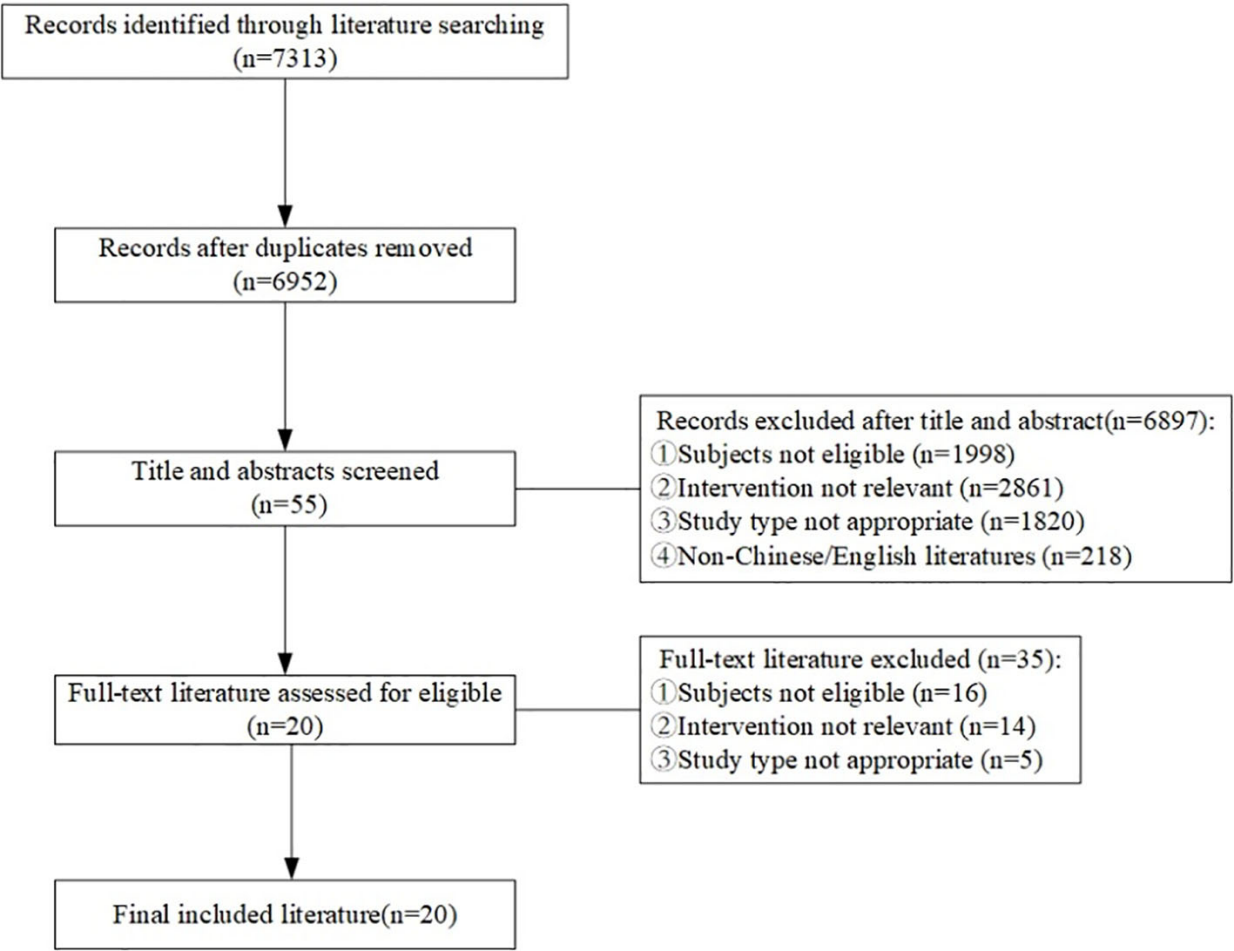
Flow diagram of literature search and selection process.

A total of 7313 articles were retrieved for this study, with 6952 remaining after removing duplicates. 55 articles were obtained after primary screening according to the titles and abstracts of these records. Finally, 20 articles were obtained after full-text reading and re-screening, including 5 clinical decisions (10–14), 5 guidelines (15–19), 3 evidence summaries (20–22), 3 recommended practices (23–25), and 4 expert consensuses (26–29). The flow diagram of the literature search and selection process is illustrated in **Figure 1**. The general characteristics of the included literature are shown in **Table 1**.

**Table 1:** Characteristics of included studies (n=20).

Author	Evidence type	Time	Literature theme	Source
Peng et al. (10)	Clinical decision	2022	Skin adverse events in molecularly targeted therapies	UpToDate
Wang et al. (11)	Clinical decision	2022	Overview of paronychia	UpToDate
Hans (12)	Clinical decision	2020	Health education for paronychia patients	UpToDate
Anthony et al. (13)	Clinical decision	2022	Prevention and treatment of paronychia	Dynamed
Shirin (14)	Clinical decision	2020	Overview of paronychia	BMJ
Zhang et al. (15)	Guideline	2019	Symptom management practice guideline: skin reaction	Cnki
Cury et al. (16)	Guideline	2020	Management guideline of cancer therapies induced dermatologic adverse events from Brazilian experts	Medlive
Lacouture et al. (17)	Guideline	2021	Guidelines for the prevention and management of skin toxicities associated with anticancer agents	ESMO
Anadkat et al. (18)	Guideline	2011	Guidelines for the prevention and treatment of EGFRIs induced skin toxicities	Pubmed
Califano et al. (19)	Guideline	2015	Management of Adverse Events from EGFR-TKI in the UK	CINAHL
Hu et al. (20)	Evidence summary	2022	Evidence summary for prevention and management of adverse skin reactions in patients with targeted therapy	Cnki
Leaderlou (21)	Evidence summary	2020	Management of EGFRIs induced skin toxicity	JBI
Sonia (22)	Evidence summary	2020	Treatment of EGFRIs induced paronychia	JBI
Guo et al. (23)	Recommended practice	2018	The recommended practice of EGFRIs induced skin toxicity	Wanfang
Lu et al. (24)	Recommended practice	2018	Evidence-based nursing for afatinib induced grade III paronychia	Cnki
Munn (25)	Recommended practice	2020	Treatment of EGFRIs induced paronychia	JBI
Potthoff et al. (26)	Expert consensus	2011	German consensus on the interdisciplinary management of EGFRIs induced skin reactions	Pubmed
Chu et al. (27)	Expert consensus	2017	Taiwanese Dermatological Association consensus for the prevention and management of EGFR-TKI related skin toxicities	Pubmed
Hu et al. (28)	Expert consensus	2019	EGFR-TKI adverse event related management Chinese Expert Consensus	Cnki
Wang et al. (29)	Expert consensus	2021	Expert consensus on management of skin toxicities induced by anti-EGFR monoclonal antibody	Cnki

EGFR-TKI means epidermal growth factor receptor tyrosine kinase inhibitor.

### 3.2 Quality evaluation results of the included studies

A total of 5 guidelines (15–19) were included in this study. The results revealed ranges for domain standardization shown in **Table 2**. A total of 4 expert consensuses (26–29) were included in this study. The evaluation results of all items were “yes,” in which the study design was complete, and the overall quality was high so that they were approved for inclusion. Currently, there is no single tool to evaluate the quality of literature for evidence summaries and recommended practices (9). Therefore, the quality evaluation was performed by tracing the references in this study. It was noted that the overall quality of the evidence summary of Hu (20), Leaderlou (21), and Sonia (22) was good, which were allowed to be included. Meanwhile, the three recommended practices, such as Guo (23), Lu (24), and Munn (25), were excellent and supposed to be included, too.

**Table 2:** Standardized scores and evaluation results in all areas of guidelines.

Included guideline	Percentage of standardization in each domain of the guide (%)						≥60% number of fields (number)	≥30% number of fields (number)	Recommended level
	Scope and purpose	Stakeholder involvement	Rigor of development	Clarity of presentation	Clarity of presentation	Editorial independence			
Zhang et al. (15)	94.44	88.89	96.88	100.00	83.30	75.00	6	6	A
Cury et al. (16)	72.22	80.56	40.63	91.97	66.67	95.83	5	6	B
Lacouture et al. (17)	83.33	77.78	50.00	70.83	58.33	50.00	3	6	B
Anadkat et al. (18)	91.67	77.78	52.08	66.67	50.00	50.00	3	6	B
Califano et al. (19)	88.89	66.67	60.42	95.83	60.00	75.00	6	6	A

### 3.3 Evidence summary and description

**Figure 2:**
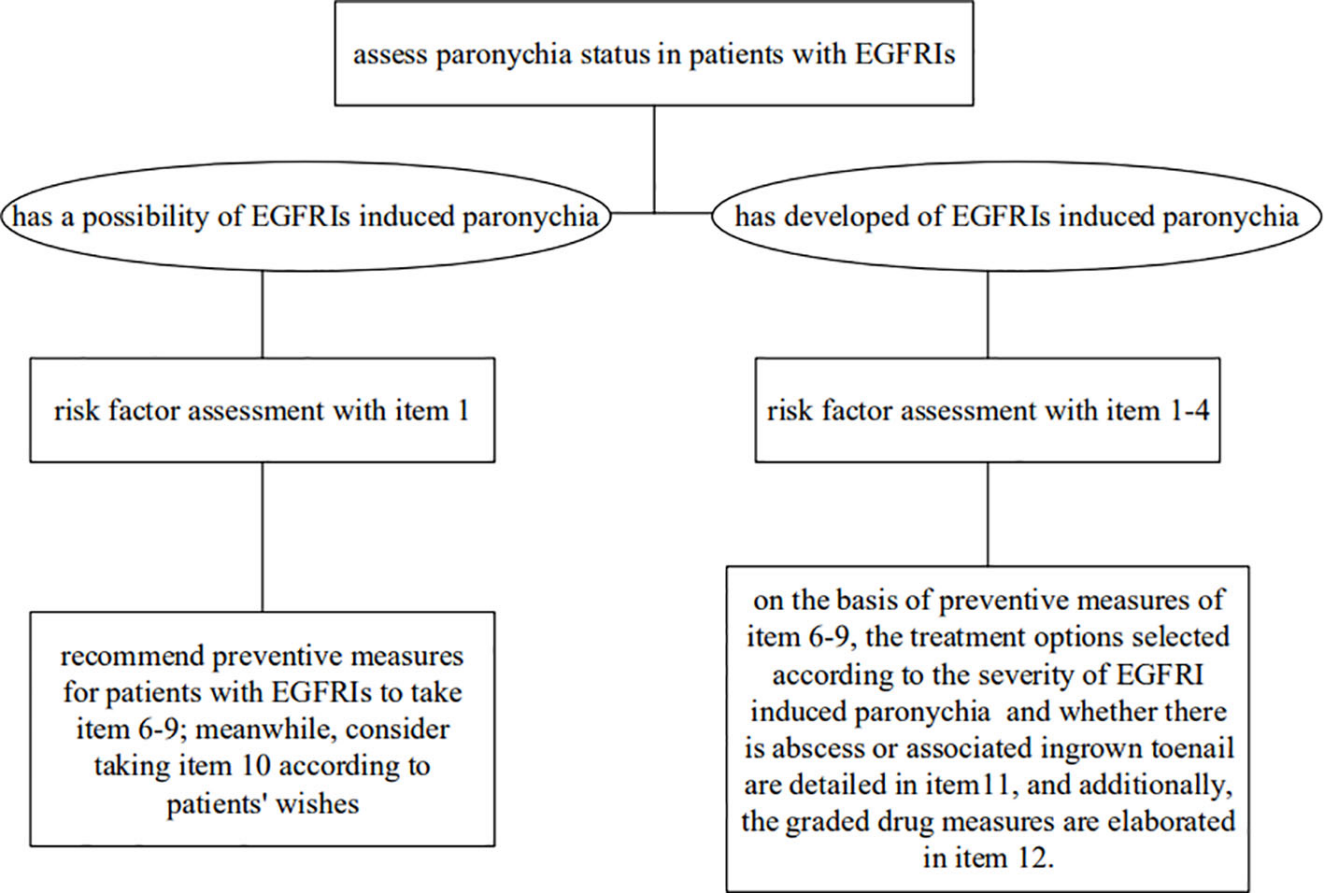
Intervention flowchart of EGFRIs induced paronychia in cancer patients.

All included evidence was graded using the Australian JBI Evidence-Based Health Care Centre Evidence Recommendation Rating System (2014 Edition) (30). According to the validity, feasibility, suitability, and clinical significance of the evidence, the recommendation level of evidence was determined as grade A or grade B based on the JBI recommendation grading. Finally, 12 pieces of relevant evidence were extracted from the included studies. These were then divided into 4 aspects: risk factor assessment, professional healthcare training, preventive measures, and therapeutic measures (**Table 3**). Intervention flowchart is detailed in **Figure 2**.

**Table 3:** Evidence summary for prevention and management of EGFRIs induced paronychia in cancer patients.

Aspects	Evidence item	Evidence level	Recommendation level
Risk factor assessment	1. Paronychia is one of the common adverse events caused by EGFRIs, and its predisposing factors include excessive manicure, nail-biting, barbing, thumb sucking, incarcerated nails, and prolonged exposure of fingers/toes to soap and water (10, 11, 13, 14).	1	A
	2. It is recommended that medical staff evaluate the location, nature, development process, symptom manifestations, severity, subjective experience of patients and reports and records of other adverse reactions of EGFRIs induced paronychia (10, 20).	5	A
	3. Digital compression test is a simple method to assist in judging paronychia abscess and its extent (11).	1	A
	4. It is recommended that the medical staffs use CTCAE, MASCC tools, or Chinese paronychia grading criteria to evaluate the severity of EGFRIs induced paronychia at least every 2 weeks. Moreover, the quality of life of cancer patients is assessed assisted using the FACT-EGFRI-18 scale (10, 20, 26, 28).	5	A
Professional healthcare training	5. Through standardized training, medical staff systematically learn relevant guidelines and expert consensus on paronychia and master the symptoms, development process, severity grading, treatment principles, and precautions of EGFRIs induced paronychia (18, 23, 27).	5	A
Preventive measures	6. It is recommended that medical staff inform cancer patients and their families of the clinical manifestations, possible impact distress, therapeutic effect, and prognosis of EGFRIs induced paronychia and instruct patients to inform them promptly when they experience symptoms (12, 20).	1	A
	7. Medical staff provide cancer patients with relevant health education and information resources to improve their knowledge literacy and self-care ability related to EGFRIs induced paronychia (23).	1	A
	8. Instruct patients to wear cotton socks and comfortable shoes to keep their hands and feet as dry as possible. Avoid sucking and biting nails; avoid contact with irritating chemicals such as acids, alkalis, and paint; and avoid nail products such as nail oils and hardeners (12, 14, 17, 22).	5	A
	9. Cancer patients are taught to correctly trim their fingers/toenails and try to round and smooth the shape of the nail margin to avoid being too short and too sharp to cause incarcerated nails (29).	5	A
	10. Creams or emollients are recommended for hand and foot moisturizing care in cancer patients. Prophylactic soaking or wet compressing of extremities with white vinegar (1:1), bleach (0.005%), boric acid (3%), iodine solution (1:10), dilute hydrochloric acid, chlorhexidine solution, povidone-iodine solution, or deionized water may be considered to inhibit microbial breeding in the hands and feet according to patients’ wishes (16, 19, 24, 27).	2	B
Therapeutic measures	11. Common paronychia treatment options include topical or oral antimicrobial agents, chemical cauterization, and partial nail extraction (10, 11, 13).	1	A
	12. It is recommended to treat EGFRIs induced paronychia graded according to the severity of paronychia. Grade 1 paronychia: soaking or wet compress with solutions such as white vinegar (1:1) and 3% boric acid is recommended (16, 19, 24, 27); topical application of topical corticosteroids, antibiotics, or bacteriostatic agents is used (11, 13, 19, 29). Grade 2 paronychia: Empirical oral antibiotics and topical beta-blockers are recommended in addition to grade 1 treatment (11, 17, 22). Grade 3 paronychia: EGFRIs dose adjustment or discontinuation is required (17, 28). Furthermore, potent glucocorticoids are used in addition to grade 2 treatment, and surgical excision of the toenails is performed if necessary (28).	1	A

## 4 Discussion

### 4.1 Scientific validity of evidence

The included studies’ quality directly influences the evidence’s accuracy and reliability. In this study, we systematically searched the relevant databases, strictly screened and evaluated the quality of the literature, and extracted and integrated the best evidence from clinical decisions, guidelines, evidence summaries, recommended practices, and expert consensus involving the prevention and management of EGFRIs induced paronychia. Two guidelines (15, 19) with all evaluation items of “yes” had a recommendation grade of A. The remaining three guidelines (16–18) had a recommendation grade of B. It indicates that the formulation process of the included guidelines was more rigorous, the methodology was reliable, and the overall quality was high. Three included evidence summaries (20–22) and three recommended practices (23–25) were found to be of good overall quality by tracing the references. All evaluation items for the 4 included expert consensuses (26–29) were “yes,” and all were high quality articles. The two researchers strictly followed the principles of rigor, transparency, science, and standardization and avoided the influence of subjective consciousness as much as possible. In the process of evidence screening, extraction, translation, and synthesis, the researchers illustrated the best current evidence in this field.

### 4.2 Clinical utility of evidence

#### 4.2.1 Risk factor assessment

EGFRIs induce keratinocyte growth arrest, differentiation, and migration disorders, thinning the periungual stratum corneum and reducing epidermal barrier integrity. Pathogens trigger periungual inflammation after entry if the nail plate punctures the nail membrane (11). EGFR plays an essential role in the normal development of the skin and its appendages, so all cancer patients receiving EGFRIs are at risk of nail changes (18). Mechanical and physical factors such as excessive manicure, nail-biting, barbing, thumb sucking, and the incarcerated nail can induce and aggravate paronychia symptoms (10, 11, 13, 14). Meanwhile, chemical injury can also destroy the nail folds barriers. Cancer patients’ fingers (toes) contacting with soap or water immersion for a long time can also induce and aggravate paronychia symptoms. Biological factors can also destroy the nail folds barriers, such as *Staphylococcus aureus*, Streptococcus pyogenes, or herpes simplex virus can also aggravate paronychia symptoms after pathogens enter the periungual tissue (10, 11, 14).

Assessment is the beginning of the symptom management process. Medical staff is advised to conduct a comprehensive assessment of the location, nature, development process, symptom manifestations, severity, subjective experience of patients, and reporting and recording of other adverse reactions to EGFRIs induced paronychia. It is recommended that medical staff strictly inquire about the onset time of paronychia, the patients’ own occupation, the presence or absence of nail painting hobbies, local exposure to substances, past medical history, medication history, and family history of nail disease (10, 20). EGFRIs induced periungual lesions are initially sterile but are prone to secondary infections such as bacterial viruses, which may lead to abscess formation. The digital compression test is a simple method to assist in judging paronychia abscess and its extent. Its primary method of operation is to apply slight pressure to the palmar aspect of the fingertip of the affected finger, which indicates the presence of an abscess if there is fading on the periungual epidermis (11). Medical staff should examine all nails with a magnifying lens in adequate light and transillumination the nails with the help of a pen lamp to locate the abnormal site. In addition, medical staff can wash the plate surface using ethanol or acetone to remove any attached material as much as possible and reduce the dazzling light to detect further minor changes in the plate surface (31). Any hemodynamic changes in the nail bed can affect the nail’s appearance. Cancer patients were instructed to relax their fingers/toes during the examination. Assessed sites included the nail plate, nail bed, proximal and lateral marginal nail folds, and subungual skin. Meanwhile, assessment content included color change, whether separated from the nail bed, nail thickness, and changes in surface texture (10, 11, 14). Besides the physical examination, it is recommended for medical staff to use CTCAE, MASCC tools, or Chinese paronychia grading criteria to evaluate the severity of EGFRIs induced paronychia at least every 2 weeks, and the quality of life of cancer patients is assessed assisted using the FACT-EGFRI-18 scale (10, 20, 26, 28).

#### 4.2.2 Professional healthcare training

With the deepening of molecular biology research, various targeted drugs have emerged, which bring hope to patients with advanced cancer. EGFRIs are prone to produce various adverse drug reactions in cancer patients during antagonizing tumor progression, which impairs the quality of life and treatment compliance of patients to some extent. Therefore, cancer patients urgently need the support and help of the medical staff. The medical staff should learn relevant guidelines and expert consensus on paronychia and systematically master the symptoms, development process, severity grading, treatment principles, and precautions of EGFRIs induced paronychia through standardized training (18, 23, 27).

Targeted drug induced periungual disease develops gradually after several weeks of medication, maybe with swelling of the lateral nail fold and tender erythematous inflammation (17). Paronychia is the most common nail change caused by EGFRIs. At the initial stage, redness, swelling, pain, and cracks began to emerge from the root margin of the nails. Afterward, there were gradual signs of inflamed ulcers on both nail folds. Later, suppurative granulation tissue gradually developed and became the incarcerated nail, eventually affecting the patient’s daily life (6, 28). Paronychia is often characterized by adverse effects such as redness, pain, swelling, exudation, bleeding, pus discharge, and irregular nail plates (14, 18, 29). Paronychia can be divided into acute and chronic depending on whether the duration of symptoms is more significant than 6 weeks (11, 13). EGFRIs induced paronychia is characterized chiefly by tender edema of multiple nail folds, which can affect both the fingers and toes of the extremities, with the thumb and the big toe occurring most frequently (11, 18, 29).

Currently, CATCAE is the most common grading standard for paronychia in clinical practice and can assist the medical staff in making scientific and reasonable clinical decisions. Grade I paronychia is mainly characterized by redness and edema of the nail fold; Grade II paronychia is mainly characterized by partial or complete detachment of the nail bed, partially affecting activities of daily living and requiring oral drug treatment; and Grade III paronychia is mainly characterized by affecting activities of daily living and requiring surgery or intravenous antibiotic treatment (22, 25, 26). A survey showed that the incidence of paronychia symptoms in cancer patients using EGFRIs ranged from 4 to 56.8%, and about 11.4% of them experienced grade 3 paronychia (19). Secondary bacterial or fungal superinfection is present in approximately 25% of patients and often presents with periungual purulent discharge (11, 17). There are various types of symptom grades in CTCAE, which are not conducive to the memory of the medical staff. The paronychia grading criteria in CTCAE can be made into an evaluation ruler and fixed on the ward wall. The treatment methods corresponding to each symptom grade can be made into a flow chart and distributed to the corresponding treatment group physicians and nurses to promote the timely and accurate evaluation, treatment, and nursing of medical staff (23).

#### 4.2.3 Preventive measures

Careful and meticulous hand and foot care can prevent the occurrence of paronychia. When using EGFRIs in cancer patients, medical staff should inform patients, and their families of the clinical manifestations, possible impact troubles, treatment effects, and prognosis of EGFRIs induced paronychia and instruct patients to inform them promptly when they experience relevant symptoms (12, 20). At the same time, medical staff can provide cancer patients with relevant health education and information resources to improve their knowledge literacy and self-care ability related to EGFRIs paronychia (23).

Avoiding various predisposing factors is essential in preventing paronychia (11). Cancer patients should avoid sucking and chewing nails; avoid contact with irritating chemicals such as acids, alkalis, and paint; and avoid nail products such as nail oils and sclerosis agents (12, 14, 17, 22). Cancer patients had better wear loose shoes and socks to avoid periungual wear and trauma (28). Occupations in which the nails are exposed to soap and water for a long time, such as chefs and cleaners, easily damage the stratum corneum or proximal nail fold and are at high risk of paronychia (11). These cancer patients should keep their hands and feet dry as much as possible, wear rubber or plastic gloves as much as possible for wading work, and not soak their hands and feet in soapy water for a long time without adequate protection (16, 19). In addition, the medical staff should teach cancer patients to trim their nails properly. The principle of ‘first middle and then two ends’ should be observed when trimming. The fingernail/toenail length shall be kept as long as possible to keep the top flush with or slightly longer than the finger/toe tip, and the nail edge shall be round and flat so as to avoid too short and too sharp to cause incarcerated nail (22, 29).

Creams or emollients can form a thin film on the skin surface after topical application, reducing water evaporation and promoting skin moisturization, thereby improving skin condition. Petroleum jelly and zinc oxide cream, and thick emollients can be recommended for hand and foot moisturizing care in cancer patients (16). Prophylactic soaking or wet compress of extremities for 15-20 minutes with white vinegar (1:1), bleach (0.005%), boric acid (3%), iodine solution (1:10), dilute hydrochloric acid, chlorhexidine solution, povidone-iodine solution, or deionized water may be considered to inhibit microbial breeding and prevent infection of hands and feet according to patients’ wishes (16, 19, 24, 27). In high-risk groups or patients who have previously experienced paronychia symptoms, it is recommended to check whether there is redness, swelling, or pain around the nails of the hands and feet every night. Furthermore, in order to prophylactically protect the periungual tissues, prophylactic use of benzalkonium chloride solution or application of compound polymyxin ointment may be considered (29).

#### 4.2.4 Therapeutic measures

Currently, treatment options for paronychia include topical or oral antimicrobial agents, chemical cauterization, and partial nail extraction (10, 11, 13). The medical staff should choose based on the severity of paronychia symptoms and the presence or absence of abscesses or incarcerated nails. If pyogenic granulomas appear in the fingers/toes of cancer patients, electrocautery silver nitrate and nail avulsion are recommended to remove excessive granulation tissue (20). In addition, warm water soaking is beneficial for digits, and biotin can help cancer patients improve nail fragility (22). If the patient feels intolerable pain, it is recommended to use non-steroidal analgesic drugs to relieve the patient’s pain (22). It is recommended that the medical staff treats paronychia graded according to the severity of EGFRIs induced paronychia.

Grade 1 EGFRIs induced paronychia: The medical staff may choose topical benzalkonium chloride and iodophor to improve erythematous edema of the tissues around the digits. Soaking or external application of white vinegar (1:1), 3% boric acid, dilute hydrochloric acid, chlorhexidine, and povidone-iodine is recommended for 15 to 20 minutes, 3 to 4 times a day (16, 19, 24, 27). If the microbial infection is suspected, a culture is required to determine the microbial status (14). Topical corticosteroids, antibiotics, or antifungal therapy were selected based on culture swab results (18). If a bacterial infection is considered, fusidic acid cream, clindamycin gel, and mupirocin ointment can be selected; if fungal infection is considered, ketoconazole cream and terbinafine cream can be selected; for cases that cannot be judged or do not have detection conditions, topical drugs that are effective for both bacterial and fungal infections can be selected such as cloiodohydroxyquine ointment (11, 13, 19, 29). Current topical microbiological agent regimens are as follows: betamethasone 0.05% + cliochinol 3% ointment, betamethasone 0.1% + gentamycin 0.05% cream, betamethasone 0.1% + gentamycin 0.1% cream, betamethasone valerate 0.1% + fusidic acid 2% cream, triamcinolone acetonide 3% + chlortetracycline 0.1% ointment or triamcinolonebenetonide 2% + fusidic acid 0.03% cream (22, 24). If the symptoms of paronychia did not improve within 2 weeks of treatment, they were treated according to the next grade (17).

Grade 2 EGFRIs induced paronychia: Empirical oral antibiotic therapy such as doxycycline, minocycline, or cephalexin, or topical beta-blockers such as betaxolol and timolol are recommended in combination with grade 1 therapeutic measures (11, 17, 22). Dicloxacillin (250 mg four times a day) or cephalexin (500 mg three to four times a day) are the most common antistaphylococcal drugs; trimethoprim-sulfamethoxazole (1-2 tablets of dual-strength tablets twice a day), clindamycin (300-450 mg four times a day), or doxycycline (100 mg twice a day) are first-line treatments for methicillin-resistant *Staphylococcus aureus* (11). If the symptoms of paronychia did not improve within 2 weeks of treatment, they were treated according to the next level (17).

Grade 3 EGFRIs induced paronychia: Potent glucocorticoids and antimicrobial agents such as clobetasol propionate 0.05%, diflumethasone valerate 0.3%, neomycin sulfate, ketoconazole cream, bifonazole cream, or terbinafine cream were continued based on the Grade 2 therapeutic measures of EGFRIs induced paronychia (28). If cancer patients developed cellulitis, intravenous antibiotics were administered for anti-infection treatment (29). The medical staff should fully evaluate cancer patients for signs of plate detachment and, if necessary, surgically remove the longitudinal and stromal segments of the fingernails or avulse the entire nail. In addition, in case of grade 3 paronychia, medical staff shall reduce the dose of the corresponding EGFRIs or suspend the treatment according to the package insert of EGFRIs. Only when EGFRIs induced paronychia has resolved to Grade 2, EGFRIs reinstate (17, 28).

## 5 Conclusion

This study systematically collates the best evidence for the prevention and treatment of EGFRIs induced paronychia in cancer patients, including 12 pieces of evidence from 4 aspects: risk factor assessment, professional healthcare training, preventive measures, and therapeutic measures, which can provide reference and reference for clinical prevention and treatment of paronychia caused by EGFRIs. Most of the recommendations in this study were Grade A, indicating that the results of this study were reliable. However, there is still a definite gap between some guideline recommendations and current clinical practice, and the generalizability of oral antibiotic strategies and patient compliance still needs to be improved. We hope that more large-sample, multi-center, and high-quality studies can be carried out in the future. A unified theoretical system and practical operation specifications can be formulated to provide more scientific and standardized guidance. The medical staff needs to comprehensively evaluate the feasibility of prevention and treatment measures for paronychia caused by EGFRIs in clinical practical application scenarios, fully consider patients’ wishes, follow the principle of individualization, and carefully apply the evidence in clinical practice. Furthermore, the medical staff should comprehensively analyze the obstacles and promoting factors of evidence application, develop targeted action strategies, implement individual and organizational changes, and root high-quality evidence in clinical practice.

## Data Availability

The original contributions presented in the study are included in the article/supplementary material. Further inquiries can be directed to the corresponding author.
